# Selected Patients With Peritoneal Metastases From Breast Cancer May Benefit From Cytoreductive Surgery: The Results of a Multicenter Survey

**DOI:** 10.3389/fonc.2022.822550

**Published:** 2022-05-11

**Authors:** Maurizio Cardi, Marc Pocard, Rea Lo Dico, Gianmaria Fiorentini, Mario Valle, Roberta Gelmini, Marco Vaira, Enrico Maria Pasqual, Salvatore Asero, Gianluca Baiocchi, Andrea Di Giorgio, Alessandra Spagnoli, Francesco Di Marzo, Bianca Sollazzo, Giuseppe D’Ermo, Daniele Biacchi, Franco Iafrate, Paolo Sammartino

**Affiliations:** ^1^Department of Surgery Pietro Valdoni, Sapienza University of Rome, Rome, Italy; ^2^University of Paris, Unité Mixte de Recherche (UMR) 1275 CArcinose et pathologies du Péritoine (CAP) Paris Tech Carcinomatosis Peritoneum Paris Technology, Digestive and Hepato-Biliary Surgery Department, Pitié Salpetrière Hospital, Assistance Publique Hôpitaux de Paris, Paris, France; ^3^University of Paris, Unité Mixte de Recherche (UMR) 1275 CArcinose et pathologies du Péritoine (CAP) Paris Tech Lariboisière Carcinomatosis Peritoneum Paris Technology, Digestive and Oncological Surgery Department, Saint Louis Hospitals, Assistance Publique Hôpitaux de Paris, Paris, France; ^4^Department of Oncology, Azienda Ospedaliera (AO) Ospedali Marche Nord, Pesaro, Italy; ^5^Peritoneal Tumors Unit, Istituto Nazionale Tumori Regina Elena, Rome, Italy; ^6^Department of Surgery, General and Oncologic Surgery Unit, Azienda Ospedaliera Universitaria (AOU) Modena, University of Modena and Reggio Emilia, Modena and Reggio Emilia, Italy; ^7^Surgical Oncology Unit, Istituto Tumori di Candiolo, Turin, Italy; ^8^Advanced Oncologic Surgery Unit, Dipartimento Area Medica (DAME) University of Udine, Azienda Sanitaria Universitaria Friuli Centrale (ASUFC) Udine, Italy; ^9^Department of Oncology, Surgical Oncology Unit, Azienda Ospedaliera di Rilievo e di Alta Specializzazione Garibaldi, Catania, Italy; ^10^Clinical and Experimental Sciences Department, University of Brescia, Azienda Socio-Sanitaria Territoriale (ASST) Cremona, Italy; ^11^Surgery of Peritoneum and Retroperitoneum Unit, Istituti Ricerca e Cura a Carattere Scientifico (IRCCS) Fondazione Policlinico Universitario A. Gemelli, Rome, Italy; ^12^Public Health and Infectious Diseases Department, Statistics Unit, Sapienza University of Rome, San Donato, Italy; ^13^General Surgery Department, Ospedale Valtiberina, Unità Sanitaria Locale (USL) Toscana Sud-Est, Sansepolcro, Italy; ^14^Department of Radiology and Oncology, Sapienza University of Rome, Rome, Italy

**Keywords:** breast cancer, peritoneal metastases, cytoreductive surgery, oligometastatic disease, ascites treatment

## Abstract

**Background:**

Even though breast cancer is the most frequent extra-abdominal tumor causing peritoneal metastases, clear clinical guidelines are lacking. Our aim is to establish whether cytoreductive surgery (CRS) could be considered in selected patients with peritoneal metastases from breast cancer (PMBC) to manage abdominal spread and allow patients to resume or complete other medical treatments.

**Methods:**

We considered patients with PMBC treated in 10 referral centers from January 2002 to May 2019. Clinical data included primary cancer characteristics (age, histology, and TNM) and data on metastatic disease (interval between primary BC and PM, molecular subtype, other metastases, and peritoneal spread). Overall survival (OS) was estimated using the Kaplan–Meier method. Univariate and multivariable data for OS were analyzed using the Cox proportional hazards model.

**Results:**

Of the 49 women with PMBC, 20 were treated with curative aim (CRS with or without HIPEC) and 29 were treated with non-curative procedures. The 10-year OS rate was 27%. Patients treated with curative intent had a better OS than patients treated with non-curative procedures (89.2% vs. 6% at 36 months, *p* < 0.001). Risk factors significantly influencing survival were age at primary BC, interval between BC and PM diagnosis, extra-peritoneal metastases, and molecular subtype.

**Conclusions:**

The improved outcome in selected cases after a multidisciplinary approach including surgery should lead researchers to regard PMBC patients with greater attention despite their scarce epidemiological impact. Our collective efforts give new information, suggest room for improvement, and point to further research for a hitherto poorly studied aspect of metastatic BC.

## Introduction

Peritoneal surface malignancies (PSMs) are a heterogeneous tumor group whose common clinical manifestation is a diffuse peritoneal involvement. These clinical conditions arise mostly from tumors in the gastrointestinal and gynecological tracts and are uncommon from primary extra-abdominal neoplasms (1). Consensus opinion typically considered patients with PSMs as having incurable disease treated by palliation alone. However, in the past 30 years, surgery for PSMs has gradually evolved according to a revised hypothesis that in selected cases PSMs are a locoregional disease that responds to a locoregional therapeutic approach (2). In recent years, several systematic reviews suggested that cytoreductive surgery (CRS) combined with hyperthermic intraperitoneal chemotherapy (HIPEC), and most recently pressurized intraperitoneal aerosol chemotherapy (PIPAC), argued against CRS as a treatment option, though it may provide survival benefits in PSMs from appendiceal, ovarian, colorectal, and gastric cancer and in malignant peritoneal mesothelioma (3–8).

Breast cancer (BC) remains among the most frequent malignancies in Western countries (9). Survival in a metastatic cohort depends on molecular subtypes, specific metastatic sites, and the disease-free interval between initial diagnosis and development of metastatic recurrence (10, 11). Although the most frequent PSMs from an extra-abdominal tumor are those from BC (PMBC) (1), clear clinical guidelines for these patients are lacking (12). Even the real incidence of PMBC is difficult to assess, and despite a well-known specific association with invasive lobular carcinoma (ILC) (13, 14), the rate varies depending on patient care settings (medical or surgical) (15) and the fact that many studies assess peritoneal involvement from BC along with other visceral metastases (16, 17).

The results in a preliminary report from investigators in our group on a small number of selected patients with PMBC treated with CRS combined with HIPEC published in 2013 (18) prompted us to gather experience from other centers dedicated to treating PSMs to evaluate how approaches for choosing these patients’ treatment options differed. Having new information on a largely neglected topic might help reappraise the surgical indications and offer to selected patients with PMBC, previously considered inoperable, a chance of cure.

Our aim in this multicenter study is to gather experience on current practice and establish whether CRS, with or without HIPEC, could be considered in selected patients with PMBC as a therapeutic option to manage abdominal spread and allow patients to resume or complete other medical treatments. As objectives to accomplish this aim, we intend to assess OS, seek possible prognostic factors, and identify metastatic patterns indicating those patients likely to benefit from curative treatment. The findings might encourage medical oncologists and surgeons not to abandon these patients tout court but to keep an open mind.

## Materials and Methods

We considered for this multicenter study only patients with a pathologically proven diagnosis of PMBC whose records included complete reliable information on the extent of peritoneal spread, identified from prospectively maintained databases in 10 referral centers for treating PSMs (9 Italian and 1 French) from January 2002 to May 2019, and retrospectively analyzed. Inclusion criteria were women between 18 and 75 years of age at PMBC diagnosis, Eastern Cooperative Oncology Group (ECOG) performance status 0–2, no more than one other metastatic site, complete clinical data on pathological and molecular subtypes, and known follow-up. Exclusion criteria were BC patients with synchronous PM and severe associated medical conditions making them fit only for palliation.

Clinical data recorded included primary cancer characteristics such as age at diagnosis, histology, TNM staging, BRCA1/2 status, type of breast cancer surgery, neoadjuvant therapy regimens, and adjuvant radiotherapy or chemotherapy or both if performed. For metastatic disease, the 10 centers were asked to report the interval elapsing between primary BC treatment and PM diagnosis, date of the patient’s admission by the referral center, molecular subtype classification, the presence and sites of other metastases, the extent of peritoneal spread, treatment aim (curative or non-curative) and type, and any other additional treatment undertaken. In the involved centers, the detailed staging depended mainly on imaging findings and included computed tomography (CT), magnetic resonance imaging (MRI), and positron emission CT (PET-CT). In each center, a multidisciplinary team (MDT) including surgeons, medical oncologists, and dedicated radiologists indicated curative or non-curative procedures (leaving *in situ* most of peritoneal metastatic burden) according to the extent of peritoneal involvement, presence of other metastatic sites, hormone status, and patients’ general conditions. Staging laparoscopy was used for histopathologic sampling or for assessing peritoneal tumor burden. Molecular subtypes were classified in all patients according to the International Expert Consensus held in St Gallen in 2013 (19). Estrogen and progesterone receptor (ER/PR) and human epidermal growth factor receptor 2 (HER2) status was determined by immunohistochemical analysis or fluorescence *in situ* hybridization following standard current guidelines (20, 21). Intrinsic subtypes were identified on metastatic peritoneal tissue sampling and when the hormone receptor or HER2 status was discordant with the primary tumor, the PMBC status was used for the analysis. Extent of peritoneal spread, recorded during laparoscopy or at surgical exploration, was evaluated using the peritoneal cancer index (PCI) according to Sugarbaker (22). Surgical technique during CRS (peritonectomy procedures) and HIPEC (cisplatin 75 mg/m^2^) for 60 min at 43°C, with the open or closed technique according to each center’s policy, has been previously described (23). In patients who underwent CRS, with or without HIPEC, residual disease was assessed using the completeness of cytoreduction (CC) score (22). Patients who underwent CRS with or without HIPEC entered the intensive care unit for at least the first 24 h after operation and received total parenteral nutrition (TPN) until oral intake became adequate. All surgical complications were evaluated according to the Clavien-Dindo classification and drug-induced toxicity was recorded and graded according to the National Cancer Institute Common Terminology Criteria for Adverse Events (CTCAE) 4.0 (24, 25). The effectiveness of strategies to control malignant ascites was evaluated according to the WHO standards, in patients subgrouped as follows: complete remission (CR), ascites disappeared for more than 4 weeks, partial remission (PR), ascites decreased by more than 50% for at least 4 weeks, stable disease (SD), ascites decreased by less than 50% or no change or progression of disease (PD), and increased ascites. The total effective rate (TER) was calculated as CR+PR/total number of cases × 100% (26). Regardless of treatment type, all patients underwent the same follow-up routine: for the first 2 years, clinical assessment every 3 months, tumor marker testing every 6 months, and diagnostic imaging every 6 months. After year 2, they underwent clinical assessment and tumor marker testing every 6 months and yearly diagnostic imaging. The institutional review board for each center approved the study procedures. Survival was calculated in months after the diagnosis of peritoneal metastases.

### Statistical Analysis

Continuous variables were summarized with descriptive statistics. Median and interquartile range were used for discrete variables, number of observations, and frequency. For comparison between groups, we used chi-square test or unpaired Student’s *t*-test, or Mann–Whitney test, as appropriate. Overall survival (OS) probabilities were estimated using the Kaplan–Meier method and displayed graphically. The log-rank test was used to compare OS groups. Univariate and multivariable data for OS were analyzed using the Cox proportional hazards model. Graphical methods to verify the proportional hazards assumption median and interquartile range included scaled Schoenfeld residuals and graphical checks proposed by Klein and Moesch Berger. Data were analyzed using R version 4.0.1 (The R Project for Statistical Computing).

## Results

Overall, in the 10 centers, 49 women with PMBC met the inclusion criteria and were considered for the study. Patients were divided into 2 groups according to the treatment intent in the involved centers. Of these 49 patients, 20 (40.8%) were treated with curative aim with CRS with or without HIPEC and 29 (59.2%) with non-curative procedures.

Patients’ clinical characteristics for the 2 groups were similar regarding primary BC, PM molecular subtype, and extent of disease according to the PCI (**Table 1**). Among the factors possibly influencing the treatment approach were poor performance status and the presence of ascites, significantly higher in the non-curative than in the curative group (*p* = 0.0005 and 0.00004). Another variable that differed in groups was the interval between BC onset and PMBC diagnosis. The median interval was significantly shorter in patients treated with non-curative intent than in the curative intent group (32 vs. 79.5 months, *p =* 0.005).

**Table 1:** Clinical characteristics in the 49 patients with peritoneal metastases from breast cancer.

Variables	Total *N* (%)	Treatment		*p*
		Curative *N* (%)	Non-curative *N* (%)	
Patients	49 (100)	20 (40.8)	29 (59.2)	ns
Age in years* (median, IQR)	56 (49–62)	56.5 (51.5–62.25)	55 (48–62)	ns
T 1	15 (30.6)	6 (30)	9 (31.1)	ns
2	32 (65.3)	13 (65)	19 (65.5)	
3	2 (4.1)	1 (5)	1 (3.4)	
N Neg	40 (81.6)	14 (70)	26 (89.7)	ns
Pos	9 (18.4)	6 (30)	3 (10.3)	
Histology				ns
Lobular	34 (69.4)	13 (65)	21 (72.4)	
Ductal	15 (30.6)	7 (35)	8 (27.6)	
BC Surgery				ns
Conservative	26 (53.1)	10 (50)	16 (55.2)	
Mastectomy	23 (46.9)	10 (50)	13 (44.8)	
Molecular Subtype				ns
Luminal A	18 (36.7)	10 (50)	8 (27.6)	
Luminal BHer2+	8 (16.3)	4 (20)	4(13.7)	
Luminal B Her2-	13 (26.6)	4 (20)	9 (31)	
Basal-like	10 (20.4)	2 (10)	8 (27.6)	
Other Metastases	0			ns
Bone	11(22.5)	5 (25.0)	6 (20.7)	
Pleura	5 (10.2)	—	5 (17.2)	
Brain	3 (6.1)	—	3 (10.3)	
Liver	1 (2.0))	–	1 (3.4)	
Interval BC-PM Median, (IQR)**	40 (26-78)	79.5 (37-132)	32 (25-45)	0.005
ECOG Score				0.0005
0	14 (28.6)	11 (55)	3 (10.3)	
1	13 (25.5)	6 (30)	7 (24.1)	
2	22 (44.9)	3 (15)	19 (65.6)	
Ascites				0.00004
Yes	22 (44.9)	2 (10)	20 (69)	
No	27 (55.1)	18 (90)	9 (31)	
Peritoneal cancer index. median (IQR)	18 (15–22)	15 (13–20.5)	20 (15–24)	ns
Completeness of cytoreduction score				ns
0	13 (26.5)	13 (65)	N/A	
1	5 (10.2)	5 (25)	N/A	
2	2 (4.1)	2 (10)	N/A	
Procedures				–
CRS+HIPEC§		13	—	
CRS§		7	—	
NIPEC°		—	8	
Palliative surgery		—	10	
HIPEC for ascites		—	6	
PIPAC^		—	5	

*Age (at diagnosis of breast cancer).

**Interval between treatment of BC and diagnosis of PM.

§Cytoreductive surgery + hyperthermic intraperitoneal chemotherapy.

°Normothermic intraperitoneal chemotherapy.

^Pressurized intraperitoneal aerosol chemotherapy.

ns, not significant; N/A, not applicable.

*Curative intent group (20 patients)*. All underwent CRS, in 13 cases combined with HIPEC. These patients were treated in only 6 of the 10 involved centers. A median 5.5 (IQR 29) month delay elapsed between PMBC diagnosis and treatment, during which 16 patients underwent a staging laparoscopy necessary for a definitive histopathologic diagnosis and PCI assessment. In 4 patients, CRS and HIPEC were originally indicated on a presumed diagnosis of ovarian cancer, based on misinterpreted cytology, tumor markers, and presence of ovarian masses. PMBC was correctly diagnosed on the surgical specimen. Two patients underwent 3 neoadjuvant chemotherapy (NACT) cycles with platinum and paclitaxel. In the other 16 patients, CRS was indicated by the MDT. Five patients had a single bone metastasis as the only extra-abdominal metastatic site, in 1 patient already present before the diagnosis of PMBC. All the patients were under medical treatment: the 16 patients with Luminal A (12) and Luminal B Her2- (4) intrinsic subtypes were under medical treatment with endocrine therapy, and the 4 with Luminal B Her2+ were under medical treatment with Her2-targeted therapy (trastuzumab). The onset of peritoneal metastases led to the development of abdominal symptoms (distension, intestinal obstruction, and pain) until worsening of the visceral crisis affecting quality of life and making it impossible to continue medical treatments. Surgery achieved complete cytoreduction (CC0) in 13 patients (65%), whereas it left residual disease in 7 patients, CC1 in 5 (25%) and CC2 in 2 (10%) patients. In the 20 patients who underwent CRS, no operative mortality arose, and 6 patients (30%) had major morbidity (**Table 2**). Two patients had an intrabdominal abscess requiring interventional radiology for drainage. Three patients needed reoperation, 2 for a postoperative intrabdominal hemorrhage, and 1 for an anastomotic leak. One patient needed ICU admission for a transient ischemic attack (TIA) medically treated without neurological sequalae. In two of the 13 patients (15.3%), HIPEC led to drug-induced toxicity, in one a grade 1 to 2 acute renal failure, in the other a grade 3 leukopenia promptly reversed with medical treatment. Of the 20 patients, 18 underwent adjuvant treatments. Indications were independently decided by each center according to the MDT, and generally based on patient’s performance status, residual disease burden, and peritoneal disease molecular subtype, different from the primary BC in 2 patients (from Luminal A to Basal-Like).

**Table 2:** Surgical procedures and outcome in the curative intent group (20 patients).

Variable	*N* (%)
Peritonectomy Procedures*	56
Pelvic	18 (32)
Anterior parietal	17 (30)
Omental bursectomy	11(20)
R/L Upper quadrant	10 (18)
Visceral Resections*	68
Greater omentectomy	20 (29)
Appendectomy	10 (15)
Hysterectomy/Adnexectomy	16 (24)
Small bowel resection	8 (12)
Right colectomy	4 (6)
Splenectomy	4 (6)
Rectosigmoid resection	3 (4)
Total gastrectomy	2 (3)
Total colectomy	1 (1)
**Outcome**	
Length of the procedure, h (median, IQR)	225 (200–272.5)
Blood loss, cc (median, IQR)	550 (300–1100)
Blood transfusions (units, *N* range)	3.2 (2–8)
ICU stay, h (median, IQR)	12 (9–18)
Postoperative stay, days (median, IQR)	15.5 (13–20.2)
**Morbidity** (grade, *N*, %, type)	
I	7 (35)
II^	7 (35)
IIIa°	2 (10)
IIIb**	3 (15)
IVa§	1 (5)

*18 patients had multiple procedures.

^4 wound infection, 1 anemia, urinary tract infection, pneumonia.

°2 intrabdominal abscess.

**2 postoperative hemorrhage, 1 anastomotic leakage.

§Transient ischemic attack.

*Non-curative group (29 patients).* Even though a shorter interval elapsed between BC and PMBC onset in the 29 patients in the non-curative group than in the curative group, a longer delay elapsed between PM diagnosis and referral to a PSM center (median 34 months, IQR 16.75). During this time, patients underwent various treatments. All the 17 Luminal A and B HER2-negative patients underwent endocrine treatment, including 2 who were subjected to bilateral oophorectomy. Endocrine suppression was achieved in 3 patients with luteinizing hormone–releasing hormone (LHRH) agonists (goserelin acetate), in 5 with aromatase inhibitors (letrozole or anastrozole), and in 9 with antiestrogens (tamoxifen). Of these 17 patients, 13 with a progressively worsening visceral crisis, involving peritoneum or other organs or both, underwent chemotherapy with anthracyclines and taxanes, combined with olaparib in BRCA-mutated patients. The 4 Luminal B HER2-positive patients underwent medical endocrine treatment with letrozole and HER2-targeted therapy with trastuzumab. The 8 Basal-Like patients underwent various systemic chemotherapy regimens, with anthracyclines, gemcitabine, or platinum-based protocols. In 20 of the 29 patients (69%), ascites led to one or multiple evacuative paracenteses. Of the 29 patients, 15 had extra-peritoneal metastases. The 6 patients with multiple bone metastases all underwent external beam radiotherapy (EBRT) for pain control, in 2 cases by 8 Gy flash and in 4 cases by a conventional hypofractionated protocol (3 Gy ×10 or 4 Gy ×5). The 3 patients with brain metastases underwent conventional standard whole brain (WB) irradiation (30 Gy ×10). Five patients had a malignant pleural effusion and underwent multiple evacuative thoracenteses. Two patients needed a tube thoracostomy after video-assisted thoracoscopic surgery and 3 underwent chemical pleurodesis with talc.

At the referral PSM center, treatment indications in these patients who had already undergone multiple different therapies depended on the dominant clinical presentation, the most frequent clinical situations being intestinal obstruction and intractable ascites. Of the 29 patients, 10 underwent palliative surgery (partial debulking, bypass, or stoma) and 19 underwent palliative treatment to control malignant ascites (6 patients laparoscopic HIPEC and 5 PIPAC) with an overall TER of 84.2% for a mean duration of 9 months (**Table 3**). The other 8 patients in stable clinical conditions underwent staging laparoscopy and a port-a-cath was implanted for long-term normothermic intraperitoneal chemotherapy (NIPEC) and to control ascites.

**Table 3:** Strategies for controlling ascites in 19 patients in the non-curative group.

	Treatment							
	Total (19)		HIPEC*(6)		PIPAC°(5)		NIPEC^ (8)	
	*N*	%	*N*	%	*N*	%	*N*	%
Complete response	10	52.6	6	100	3	60	1	12.5
Partial response	6	31.6	—	—	1	20	5	62.5
Stable disease	3	15.8	—	—	1	20	2	25
Progressive disease	—	—	—	—	—	—	—	—
Total effectiveness rate	16	84.2	6	100	4	80	6	75
Ascites control (mean, months)	9		13		5		2	

*HIPEC, Hyperthermic intraperitoneal chemotherapy.

°PIPAC, Pressurized intraperitoneal aerosol chemotherapy.

^NIPEC, Normothermic intraperitoneal chemotherapy.

**Figure 1:**
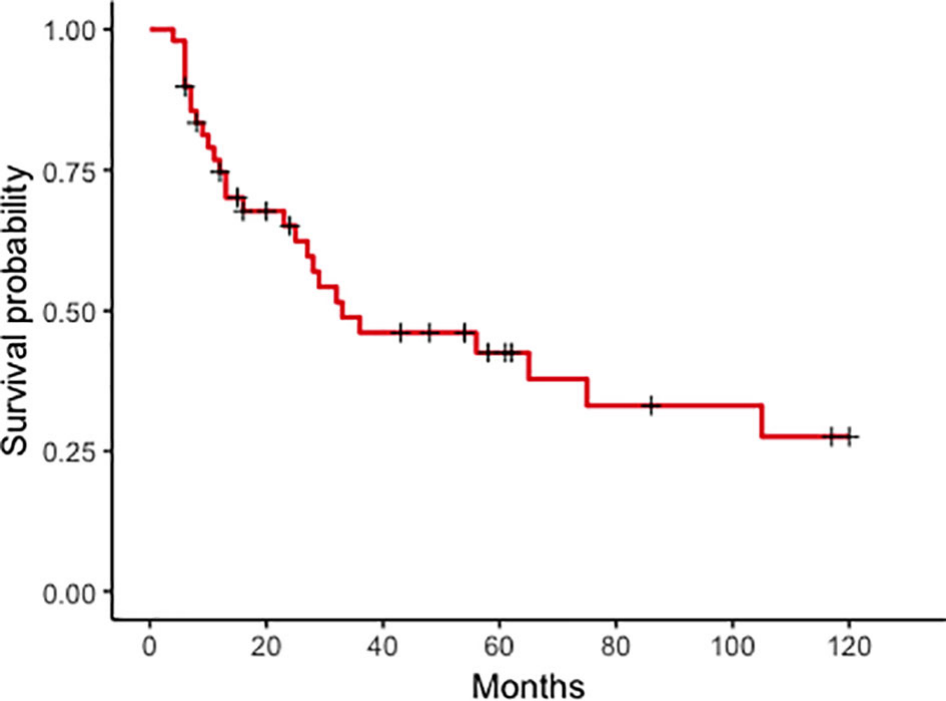
Peritoneal metastases from breast cancer. Kaplan–Meier curves plotted for overall survival in 49 patients with peritoneal metastases from breast cancer at 120 months.

**Figure 2:**
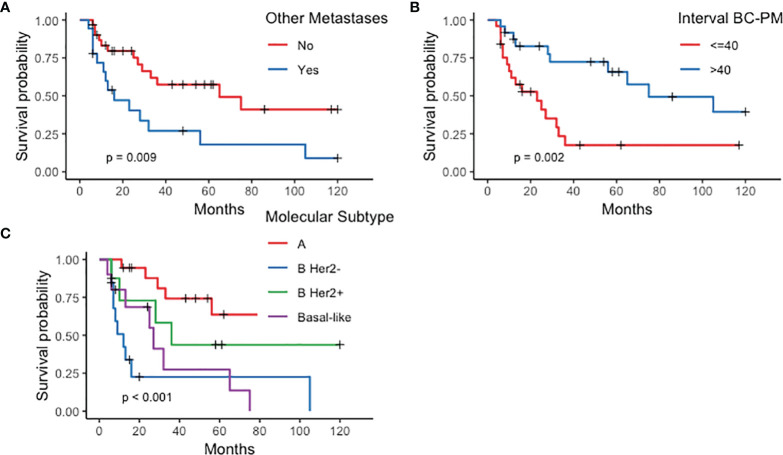
Kaplan–Meier curves plotted for prognostic factors significantly influencing survival in patients with peritoneal metastases from breast cancer (PMBC). **(A)** Other metastases; **(B)** Interval between breast cancer (BC) and peritoneal metastases (PM) diagnosis; **(C)** molecular subtype.

*Survival and prognostic factors*. Median follow-up after PM treatment was 61 months (range 6–120). The 10-year OS rate was 27% (95% CI: 0.15–0.51) (**Figure 1**), with a median 33-month survival. Median OS in the curative group was 61.5 months, with a significantly better OS than patients treated with non-curative procedures (89.2%, 95% CI: 30.8–92.36% vs. 6%, 95% CI: 0.92–39 at 36 months, *p* < 0.001). After CRS, 13 patients had recurrent or progressive disease (peritoneal or extra-peritoneal) at a median 54-month interval. Seven (35%) had a peritoneal recurrence, alone in 4 cases or combined with other sites in 3, after a median of 39 months. Risk factors significantly influencing survival at univariate analysis were molecular PM subtype, interval between primary BC treatment and diagnosis of PM, and presence of extra-peritoneal metastases (**Figure 2**). Multivariate analysis confirmed that factors significantly influencing survival were age at primary BC treatment, molecular subtype classification, and presence of other distant metastases (**Table 4**).

**Table 4:** Prognostic factors influencing survival: univariate and multivariate analysis in the 49 patients with peritoneal metastases from breast cancer.

Variable	Univariate			Multivariate		
	HR	95% CI	*p*-value	HR	95% CI	*p*-value
Molecular subtype						
Luminal A						
Luminal B Her 2-	8.16	2.69–24.83	0.0002	13.59	3.50–52.79	<0.0001
Luminal B Her 2+	2.39	0.64–8.91	0.1952	7.80	1.60–38.09	0.011
Basal-Like	5.01	1.61–15.51	0.0051	2.43	0.72–8.22	0.151
Other metastases						
No						
Yes	2.653	1.24–5.67	0.0118	4.48	1.62–12.36	0.004
AGE	1.03	0.99–1.07	0.105	1.07	1.02–1.12	0.002
Interval BC-PM*						
≤40 months						
>40 months	0.29	0.13–0.67	0.00361	0.48	0.15–1.49	0.21

*Interval between breast cancer (BC) and peritoneal metastases (BM).

## Discussion

Rather than comparing incomparable groups, we designed this study to take a snapshot of current treatment in patients with PMBC to identify those who might benefit from CRS. Even though we analyzed data for patients with PMBC whose extent of disease (peritoneal or extraperitoneal) and therefore therapeutic options and outcomes differed, our findings now extend the extremely limited previously published information on this topic (15) and possibly suggest that few selected patients can undergo curative CRS rather than non-curative procedures and obtain unexpectedly favorable OS.

A strong point in our study is that the multicenter cooperation enabled us to collect a sufficiently large group for statistical analysis. Similar findings for comparison are hard to find. For example, Flanagan et al. (1) reporting their over 18 years’ experience in a major European center for PSM treatment reported an extremely poor outcome in 22 patients with PMBC, with 3.8% of all cases observed in the same period with a median OS rate of only 5.8. months. Apart from a single case report (27, 28), others who analyzed case series including numerous patients with PMBC reported an outcome after hormone or chemotherapy treatments that ranged from a median OS of 1.5 to 19 months (13, 29, 30). In a small series analyzing PMBC exclusively involving gynecological areas, surgical excision combined with complete CRS achieves a median OS of 34 to 36 months (31, 32). Last, in recent years, an international series analyzing data for patients treated with CRS combined with HIPEC for PMS originating from unusual cancer sites and analyzing a total 734 cases included 17 patients (2.3%) with PMBC treated with CRS without specifying patients’ features, and reporting a high HR for OS (33).

In our series, in substantial agreement with published findings (10, 11, 34), statistically significant prognostic variables are the disease-free interval elapsing between primary tumor and metastatic disease, age, and molecular subtype. In the natural history of BC, metastatic disease develops in about 20% of patients overall (35), 5-year survival reaching 25% (36, 37) with only 2%–5% of patients surviving at least 10 years (38, 39).

What is prognostically especially important are the metastatic sites involved (10, 34), frequently reported as depending on the molecular subtype (16, 17), given that patients with bone involvement alone have far better outcomes than those with visceral metastases (40, 41). The presence of visceral metastases and their progression over time lead to the so-called visceral crisis, defined according to ESO/ESMO guidelines (36) as a severe organ dysfunction causing clinical conditions to worsen rapidly, thus hindering ongoing management to seek a valid therapeutic alternative (42, 43), as happened in our patients. The development of peritoneal metastases during medical treatment led to various degrees of intestinal obstruction, with abdominal discomfort and pain, causing ongoing medical treatments to be suspended, leaving the patients with no alternatives but surgery. Obviously, in these clinical conditions, CRS could be considered as an option only in patients promptly referred to a PSM specialized center, with a low PCI and with no or minimal extraperitoneal disease. As a therapeutic approach to cope with these dramatically worsening clinical events, our findings tend to support the importance of surgery in more aggressive multidisciplinary approaches in selected patients with PMBC, similarly to what some report for other visceral metastases from BC (43) given that in recent years and under certain conditions, the surgical indications for metastatic BC have widened to include the abdomen and other districts (44–49).

In our multicenter series of patients with PBMC, when we investigated factors related to outcome, the best results correlated with a lengthy interval between BC and PBMC onset, positive HR status, possible treatment with HER2-targeted agents, and with PM as the only metastatic location. In 15 of the 20 patients treated with curative intent, the only metastatic site was the peritoneum and the remaining 5 patients besides PM had a single bone metastasis. Treating metastatic disease from BC with locoregional therapy seems justified beyond specific emergency conditions (visceral crisis) also because some patients have limited rather than extensive disease both as number of sites detected and as metastatic burden, an oncologic circumstance known as oligometastatic disease, a concept first proposed by Hellman et al. (50). Locoregional therapy for BC oligometastases accords with international guidelines (36, 51) and has in recent years been reappraised in a Dutch metanalysis that proposed including, among the indications for a multidisciplinary approach, a single metastatic site (52). Others document long-term survivals and a possibility of a cure in numerous patients with a single metastatic site from BC treated with locoregional therapy (radiotherapy or surgery) associated with systemic therapy especially those with a low tumor burden (53).

When we investigated current practice with locoregional therapy in oligometastatic BC, we found that the only data available concern liver or lung metastases because they have greater epidemiologic frequency than PMBC (49, 54, 55). Its minor incidence has so far prevented researchers from directly applying these approaches to PMBC. The study conducted in recent years by Chun et al. (49) shows that in patients with liver metastases from BC, surgical excision followed by systemic therapy yielded higher OS rates than systemic therapy alone. Although we cannot directly compare these results with those in our PMBC patients who underwent CRS, because we lack a homogenous class for comparison, our OS rate in resected patients overlaps that reported by American investigators even though the different molecular subtypes result in different outcomes.

When we compared clinical history and outcomes in our patients treated with curative versus non-curative intent, several new findings emerged. Unlike those who underwent curative surgery at the referral center, the 29 patients who underwent non-curative treatments for PMBC in 15 cases had, apart from the peritoneum, extensive involvement elsewhere. Besides, patients in whom the peritoneum was the only metastatic site had widespread disease and severe ascites. These patients, unsuitable for CRS, underwent surgery aimed exclusively to resolve intestinal obstruction and treat ascites, both achieving modest survival. In these patients, and particularly in those with initial peritoneal involvement alone, one reason jeopardizing possible CRS is the significant time lapse between PM diagnosis and referral to an experienced PSM center (median 34 months versus 5.5 months in the curative group) probably reflecting the commonly held concept of untreatable PMBC. The TER in controlling malignant ascites with different palliative treatments in 19 patients was 84% for a mean duration of 9 months, varying from 100% for HIPEC to 75% in patients who underwent NIPEC. The few patients treated make it difficult to draw conclusions on the best strategy.

Nevertheless, when we sought more information indicating those patients with PBMC most likely to benefit from curative treatment, our findings indicate that in a patient operated on for BC at the onset of oligometastatic disease involving the peritoneum alone with a low PCI, we should at least consider CRS, especially in Luminal A patients in whom a lengthy interval elapsed between primary cancer treatment and PMBC diagnosis. Even though, as happens also in other clinical conditions commonly coexisting with PSM, intraperitoneal recurrence reached 35% of the patients who underwent CRS, this procedure achieved a median 61-month OS, among the highest reported in a peritoneal metastatic disease setting (3).

Conversely, our small series prevents us from stating whether combining CRS with HIPEC influenced our patients’ outcomes. In a hormone-sensitive neoplasia like BC, HIPEC probably has minor therapeutic value, and could also unjustifiably increase morbidity.

Among the study limitations is the lack of a centralized pathological review regarding HR and HER2 status as well as Ki67. Our decision to classify molecular subtypes on IHC surrogates rather than undertaking genomic testing slightly reduced accuracy for identifying BC subtypes. We nevertheless underline the need to ascertain these variables in samples from PMBC owing to possible discordance between the primary tumor and metastases (56).

## Conclusions

Current practice generally considers patients with metastatic BC incurable, and treatment aims to control tumor burden and improve symptoms and quality of life (57). In the past decade, although several drugs have been available for patients with metastatic BC, the increase in survival is minimal (58). The significantly higher increase in the frequency of visceral metastases from BC over the past 10 years (39) and the improved outcome in selected cases after a multidisciplinary approach including surgery (59) should lead researchers to regard PMBC patients with greater attention despite their scarce epidemiological impact. The results of our survey show that surgery with a curative intent could be considered in selected patients with Luminal A, no extraperitoneal disease, and in whom a lengthy interval elapsed between primary cancer treatment and peritoneal metastases diagnosis. Although these findings gathered from a limited experience hardly offer a sufficient basis to provide general guidelines, our collective efforts give new information, suggest room for improvement, and point to further research for a hitherto poorly studied aspect of metastatic BC. Despite their limited epidemiological impact, these retrospective results on PMBC merit confirmation in future prospective studies.

## Data Availability Statement

The original contributions presented in the study are included in the article/supplementary material. Further inquiries can be directed to the corresponding author.

## Ethics Statement

Ethical review and approval was not required for the study on human participants in accordance with the local legislation and institutional requirements. Written informed consent for participation was not required for this study in accordance with the national legislation and the institutional requirements.

## Author Contributions

MC and PS: original design of the study and writing of the original draft. MP, RD, GF, MVal, RG, MVai, EP, SA, GB, AG, G. D’E, and DB: data acquisition and collection. AS: statistical analysis. FI: diagnostic imaging review. FM and BS: literature search and data analysis. All authors critically reviewed the draft and approved the final version.

## Conflict of Interest

The authors declare that the research was conducted in the absence of any commercial or financial relationships that could be construed as a potential conflict of interest.

## Publisher’s Note

All claims expressed in this article are solely those of the authors and do not necessarily represent those of their affiliated organizations, or those of the publisher, the editors and the reviewers. Any product that may be evaluated in this article, or claim that may be made by its manufacturer, is not guaranteed or endorsed by the publisher.
